# Methylation sites of human papillomavirus 16 as potential biomarkers for cervical cancer progression

**DOI:** 10.3389/fonc.2025.1481621

**Published:** 2025-01-27

**Authors:** Sha Ji, Nannan Ji

**Affiliations:** ^1^ Department of Gynecology, Xinjiang Uygur Autonomous Region People’s Hospital, Urumqi, Xinjiang, China; ^2^ Operating Room, The First Affiliated Hospital of Shihezi University, Shihezi, Xinjiang, China

**Keywords:** human papillomavirus 16, cervical cancer, methylation, biomarkers, pyrosequencing

## Abstract

**Objective:**

To investigate the methylation levels at 13 specific sites of the human papillomavirus 16 (HPV16) L1 gene as potential biomarkers for the diagnosis of cervical cancer.

**Methods:**

Samples were collected from the gynecological outpatient and inpatient departments of the Xinjiang Uygur Autonomous Region People’s Hospital. A total of 107 women participated in this study, including 54 with cervical cancer (32 Uygur, 22 Han) and 53 with cervical inflammation (32 Uygur, 21 Han). Methylation analysis was performed using pyrosequencing to quantitatively assess methylation levels at specified CpG sites within the HPV16 L1 gene.

**Results:**

High methylation levels were predominantly observed at sites 5927, 5963 and 6367 in cervical cancer cells compared with inflammatory cells. Methylation patterns exhibited no significant differences between the Han and Uygur ethnic groups but correlated with viral load and age within each group. Receiver operating characteristic curve analyses of these methylation sites indicated high diagnostic accuracy in distinguishing between high-grade lesions and less severe conditions.

**Conclusions:**

Methylation of specific CpG sites in the HPV16 L1 gene holds promise as a biomarker for cervical cancer progression. The gene locus at position 6367 has important features in the methylation pattern of cervical cancer, and high accuracy shown in diagnosis make it a potential biomarker for early diagnosis of cervical cancer.

## 1 Introduction

Persistent infection with high-risk human papillomaviruses (HR-HPVs) and the subsequent up-regulation of viral oncoproteins E6 and E7 are recognized as critical molecular events in cervical carcinogenesis (1, 2). These oncoproteins interfere with the function of key host tumor suppressor proteins, leading to malignant transformation. Specifically, E6 promotes the degradation of p53, a tumor suppressor essential for programmed cell death, whereas E7 inactivates the retinoblastoma protein (pRb) that normally regulates cell cycle progression (3, 4). This disruption of p53 and pRb functions results in chromosomal instability and cancer development (5). Among the various types of HR-HPVs, HPV16 is the most prevalent (followed by HPV18) and contributes to over 50% of cervical cancer cases worldwide (6–8).

Infection with HPV occurs in the undifferentiated basal cells of the cervical epithelium, where the viral early proteins E1, E2, E6 and E7 are expressed at low levels (9). As the infected cells differentiate, viral late proteins L1 and L2 are produced for capsid formation and viral particle assembly. The E4 protein assists in the release of viral particles by associating with the host cell cytoskeleton (10, 11). The production of the highly immunogenic L1 protein is regulated by host proteins and epigenetic modifications, ensuring that it is only expressed in differentiated cells, thus evading immune detection (12). The HPV16 L1 protein and its associated mRNA are detectable in low-grade cervical lesions and productive infections, but its absence is highly correlated with high-grade lesions (13, 14). Although the L1 coding sequence remains intact in transformed cells, capsid proteins are not synthesized (15).

Although HR-HPV infection is a necessary precursor to cervical cancer, only a small fraction of infections among women progress to cancer (16, 17). Current HPV DNA testing is not sufficiently specific to accurately identify women who are HR-HPV positive who require colposcopy, as many infections are transient (18). Genotyping for HPV16 and HPV18, combined with cytology, is currently recommended for cervical cancer screening (19); however, more specific biomarkers are needed to triage women who are HPV16 or HPV18 positive and reduce unnecessary colposcopy referrals (20, 21).

The methylation of both host and HPV genes has been extensively studied and shown to be associated with cervical abnormalities (22, 23). Methylation modifications, such as CpG site methylation within the L1 gene, can control the expression of this gene, which is often silenced in transformed cervical cells. High methylation levels in the 3’L1 gene region have been reported through bisulfite sequencing, indicating a potential role in controlling L1 expression (24, 25); however, methods such as bisulfite sequencing and direct sequencing can lead to inaccurate methylation level estimations in clinical samples. Pyrosequencing, a more accurate quantification method, has been employed to measure HPV DNA methylation, revealing hypermethylation in the L1 and L2 regions of various HPV types (26, 27). Recent studies suggest that L1 gene methylation can distinguish between cervical intraepithelial neoplasia 3 (CIN3) and invasive cervical carcinoma (26, 28).

This study assesses the methylation levels of the HPV16 L1 gene across 13 specific CpG sites (5927, 5963, 6367, 6389, 6457, 6581, 6650, 6731, 6797, 7034, 7091, 7136 and 7145) in cervical samples from Uygur and Han women. By comparing methylation patterns in samples from patients with cervical cancer with those from patients with cervical inflammation, we seek to identify potential biomarkers for the early diagnosis and prognosis of cervical cancer. This is particularly relevant for the Uygur population, where high cervical cancer incidence contrasts with lower HPV infection rates (29, 30). Understanding the methylation landscape of the HPV16 L1 gene could enhance screening protocols, enabling more precise identification of women at high risk for rapid cancer progression.

We used pyrosequencing to quantitatively analyze the methylation levels of the selected CpG sites. This method offers high accuracy and sensitivity, making it suitable for clinical applications. The study included 54 patients with cervical cancer (32 Uygur, 22 Han) and 53 patients with cervical inflammation (32 Uygur, 21 Han). Our goal was to determine the correlation between methylation levels and clinical parameters, such as viral load and patient age, within each ethnic group. We also aimed to identify any significant differences in methylation patterns between the two groups.

The findings indicate that, although there are no significant differences in methylation levels between the Han and Uygur groups, specific CpG sites within each group show strong correlations with clinical parameters. These results suggest that HPV16 L1 methylation could serve as a valuable biomarker for cervical cancer progression, especially in populations with unique epidemiological patterns, such as the Uygur population.

In summary, this study underscores the potential of HPV16 L1 gene methylation as a biomarker for cervical cancer. Further research involving larger cohorts is necessary to validate these findings and refine the use of methylation levels in clinical practice.

## 2 Materials and methods

### 2.1 Clinical samples and human papillomavirus 16 DNA detection

Cervical swabs from patients at the gynecological outpatient and inpatient departments of the Xinjiang Uygur Autonomous Region People’s Hospital were collected between September 2012 and October 2014. The samples included 54 specimens from patients with cervical cancer (32 Uygur, 22 Han) and 53 specimens from patients with cervical inflammation (32 Uygur, 21 Han). All participants provided informed consent, and the study was approved by the Ethics Committee of Shihezi University.

Human papillomavirus DNA detection was performed using the Genomica CLART^®^ HPV2 test (Spain) in the virology unit of the microbiology department of Shihezi University. Samples positive for HPV16 were identified, and only these were included in the methylation analysis. Two HPV16-positive cell lines, CaSki (CRL-1550) and SiHa (HTB-35), were purchased from ATCC (VA, USA) and used as positive controls for amplification and pyrosequencing.

### 2.2 Bisulfite conversion and methylation analysis via pyrosequencing

The HPV16 DNA-positive samples underwent bisulfite conversion using the EZ DNA Methylation-Gold Kit (Zymo Research, CA, USA) following the manufacturer’s protocol. Deoxyribonucleic acid (100–1,000 ng) was extracted from the clinical samples and modified using bisulfite treatment to convert unmethylated cytosines to uracils while leaving methylated cytosines unchanged. The modified DNA was stored at –20°C until further analysis.

Specific primers for the 13 CpG sites within the HPV16 L1 gene were designed using MethPrimer software (http://www.urogene.org/methprimer/). The forward (FW) and reverse (RV) primers for the amplification of these sites are as follows:

- FW: 5’–biotin–GGTTAAATTAAAATTTATATTAGGAAAA–3’ and RV: 5’–AAACATATACACAACAAACAACACTAATTC–3’ (140 bp) for CpG sites 5927 and 5963.
- FW: 5’–biotin–TAATATATAATTATTGTTGATGTAGGTGAT–3’ and RV: 5’–AACAATAACCTCACTAAACAACCAAAA–3’ (130 bp) for CpG sites 6367, 6389, 6457, 6581, 6650, 6731, 6797, 7034, 7091, 7136 and 7145.

Polymerase chain reaction (PCR) amplification was performed using the bisulfite-modified DNA. The reaction mixture contained 13.6 μL of DNase/RNase-free water, 1 × PCR buffer, 2.5 mM MgCl2, 250 μM dNTPs, 12.5 pM of each primer and one unit of HotStart HiFidelity DNA polymerase (Affymetrix, MA, USA). The thermal cycling conditions were as follows: initial denaturation at 95°C for 10 minutes, followed by 50 cycles of 95°C for 30 seconds, 55°C for 1 minute and 72°C for 1 minute, with a final extension at 72°C for 10 minutes. The PCR products used were verified via 1.5% agarose gel electrophoresis.

Prior to pyrosequencing, 20 μL of the biotin-labelled PCR products were purified using Streptavidin Sepharose High-Performance beads (GE Healthcare, IL, USA), denatured and mixed with 0.4 μM of sequencing primers. The samples were then loaded onto a PyroMark^™^ Q24 Advanced System (Qiagen, Germany) for sequencing.

### 2.3 Statistical analysis

Statistical analyses were performed using SPSS version 17.0 software (IBM, Armonk, NY, USA). Differences in methylation levels among different groups were analysed using the Kruskal–Wallis test. Receiver operating characteristic (ROC) curve analysis was employed to determine the sensitivity and specificity of methylation levels at various cutoff points to differentiate between the different stages of cervical lesions (≤CIN3 vs squamous cell carcinoma [SCC] and ≤CIN2 vs CIN3+). The area under the ROC curve (AUC) values were calculated using ROC curves, which were first plotted on a graph with false positive rates as abscissa and true positive rates as ordinate. A *p*-value <0.05 was considered statistically significant.

This comprehensive analysis aimed to identify potential methylation biomarkers within the HPV16 L1 gene that could be used for the early detection of cervical cancer, particularly in high-risk populations.

## 3 Results

### 3.1 Methylation levels of the human papillomavirus 16 L1 gene at 13 CpG sites

Methylation levels were assessed at 13 specific CpG sites within the HPV16 L1 gene: 5927, 5963, 6367, 6389, 6457, 6581, 6650, 6731, 6797, 7034, 7091, 7136 and 7145. The study included samples from a combined cohort of women from Han and Uygur populations. The methylation patterns of the HPV16 L1 gene in these clinical samples exhibited a range of methylation percentages, with notable variability across different CpG sites. Average methylation levels across the 13 sites ranged from 16.9% at site 6581 to 35.9% at site 6367, highlighting significant heterogeneity in methylation patterns (**Table 1**). This variability underscores the potential of the methylation of specific CpG sites as biomarkers for cervical cancer progression, suggesting the value of a comprehensive approach to further explore their diagnostic and prognostic value.

**Table 1:** Descriptive statistical analysis of the methylation rates at 13 CpG sites in cervical cancer patients from Han and Uygur populations.

Gene locus	N	M ± QR
5927	54	34.9 ± 24
5963	54	32.7 ± 20.3
6367	54	35.9 ± 23.3
6389	54	33.2 ± 19.0
6457	54	30.7 ± 16.3
6581	54	16.9 ± 12.0
6650	54	31.0 ± 12.5
6731	54	27.5 ± 17.3
6797	54	25.8 ± 15.3
7034	54	28.4 ± 12.0
7091	54	27.7 ± 9.0
7136	54	23.4 ± 9.0
7145	54	25.9 ± 8.3

Methylation levels at the 13 CpG sites in the samples from the patients with cervical inflammation from the Han and Uygur populations were analysed separately and are presented in **Table 2**. The data includes comparisons between cervical inflammation and cervical cancer within both ethnic groups. For both groups, methylation levels were significantly higher in cervical cancer samples than in cervical inflammation samples. **Tables 3** and **4** illustrate the statistically significant differences in methylation levels between cervical inflammation and cervical cancer within each ethnic group (*p* < 0.001). In the Han population, all loci except for 6457 and 6581 loci showed significant differences in methylation levels between cervical inflammation and cervical cancer. In the Uygur population, all loci showed significant differences in methylation levels between cervical inflammation and cervical cancer (*p* < 0.001).

**Table 2:** Descriptive statistical analysis of methylation rates at 13 CpG sites in cervical inflammation patients from the Han and Uygur population.

Gene locus	N	M ± QR
5927	53	22.3 ± 8.8
5963	53	20.1 ± 13.8
6367	53	21.1 ± 18.6
6389	53	19.3 ± 13.2
6457	53	16.7 ± 18.3
6581	53	11.3 ± 5.2
6650	53	15.1 ± 20.0
6731	53	16.3 ± 13.2
6797	53	13.7 ± 11.0
7034	53	13.9 ± 19.0
7091	53	13.3 ± 17.0
7136	53	14.2 ± 11.6
7145	53	15.3 ± 15.8

**Table 3:** Comparison of cervical inflammation and cervical cancer within the Han population.

Gene locus	Z	P
5927	-3.13	0.001
5963	-3.13	0.002
6367	-4.231	0.000
6389	-3.48	0.001
6457	-0.113	0.910
6581	-0.575	0.056
6650	-3.49	0.000
6731	-3.12	0.002
6797	-4.06	0.000
7034	-3.6	0.000
7091	-3.5	0.000
7136	-3.5	0.001
7145	-4.12	0.002

**Table 4:** Comparison of cervical inflammation and cervical cancer within the Uygur population.

Gene locus	Z	P
5927	-5.096	0.000
5963	-5.1	0.000
6367	-5.2	0.000
6389	-5.5	0.000
6457	-5.6	0.000
6581	-5.09	0.000
6650	-5.6	0.000
6731	-5.4	0.000
6797	-5.2	0.000
7034	-5.16	0.000
7091	-4.5	0.000
7136	-5.1	0.000
7145	-5.4	0.000

For classification purposes, methylation levels were categorized into high (>60%), intermediate (20%–60%) and low (<20%) based on control cell lines and clinical sample distributions. High methylation levels were observed prominently in CpGs 5927, 5963, 6367 and 6389 in SCC samples, whereas normal and CIN1 samples mostly exhibited low methylation levels. In SCC samples, CpG sites 5927 and 5963 showed high methylation in 100% and 90% of cases, respectively, indicating a strong correlation with malignant lesions.

### 3.2 Potential biomarkers of specific CpG site hypermethylation in the human papillomavirus 16 L1 gene for the prediction of cervical cancer

To determine the utility of the methylation of specific CpG sites as biomarkers for cervical cancer progression, we performed ROC curve analyses, evaluating the sensitivity and specificity of methylation levels at various cutoff points (10%, 15%, 20%, 25% and 30%) for predicting lesion severity (CIN3+ and SCC).

**Figure 1:**
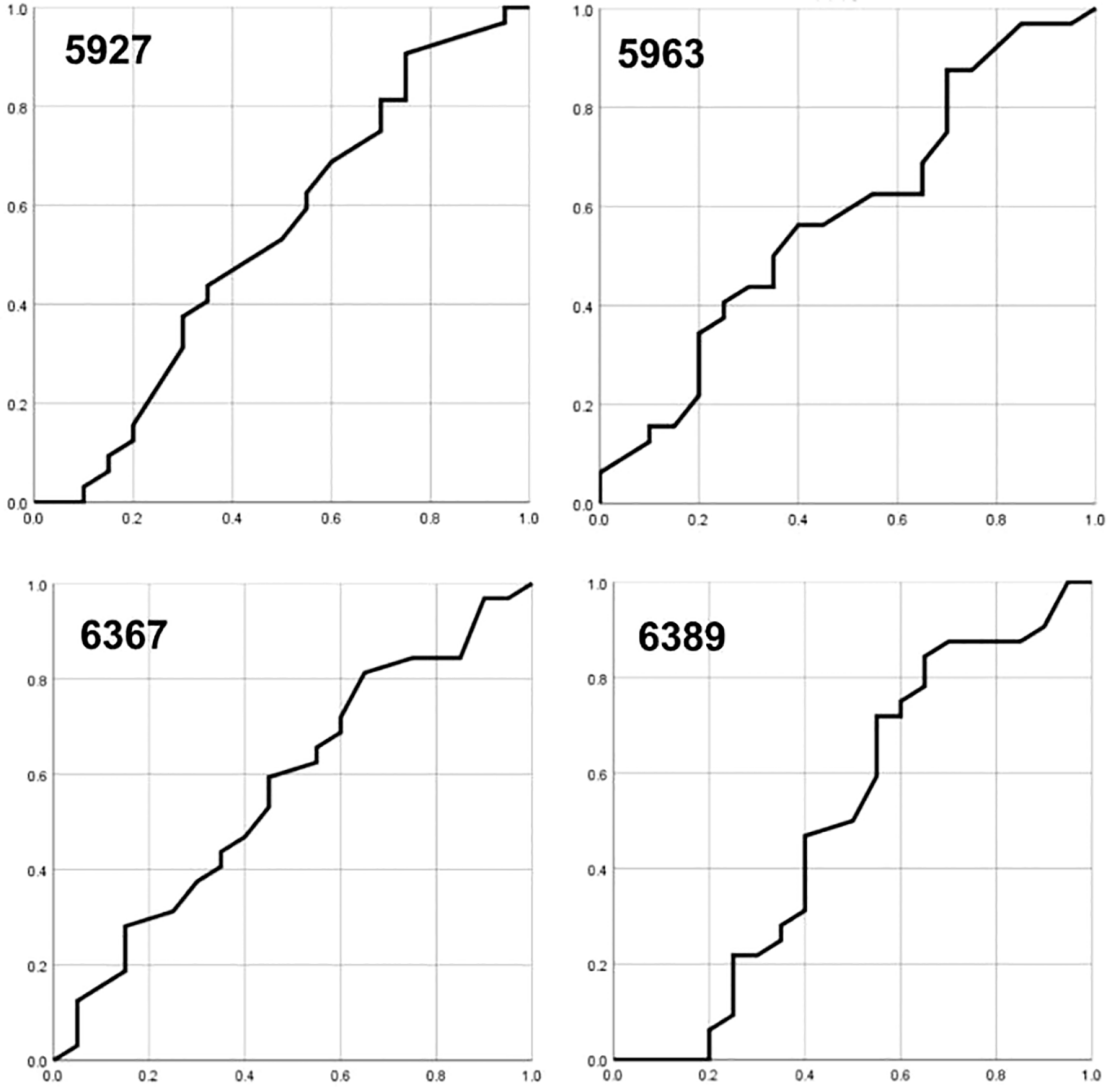
The area under the ROC curves (AUC) for CpG sites 5927, 5963, 6367, and 6389, respectively.

The AUC for CpG sites 5927, 5963, 6367 and 6389 ranged from 0.92 to 0.95, indicating high diagnostic accuracy (**Figure 1**). Specifically, CpG sites 5927 and 5963 demonstrated superior sensitivity and specificity to other sites at different methylation cutoffs. A cutoff of 20% methylation provided optimal balance, distinguishing high-grade lesions (CIN3+ and SCC) with high sensitivity and specificity.

Notably, combining the methylation data from CpG sites 5927 and 5963 at a 15% cutoff point increased both sensitivity (100%) and specificity (97%), suggesting a robust biomarker profile for cervical cancer progression. These results highlight the potential of hypermethylation at specific CpG sites within the HPV16 L1 gene as predictive biomarkers for cervical cancer, warranting further validation in larger cohorts.

Overall, our findings indicate that the methylation levels at specific CpG sites in the HPV16 L1 gene can serve as reliable biomarkers for the early detection of cervical cancer. High methylation levels, particularly at CpG sites 5927 and 5963, are strongly associated with advanced cervical lesions, making them valuable targets for clinical screening and diagnostic protocols. Further studies are needed to refine these biomarkers and explore their application in diverse populations.

## 4 Discussion

The role of HR-HPVs in the progression of cervical cancer is well documented and highlights the critical involvement of HPV, especially types 16 and 18, which are implicated in over half of all cervical cancer cases worldwide. However, the natural history of HPV infection includes many transient cases, meaning only a minority of HR-HPV infections evolve into cancer. This underscores the need for precise biomarkers beyond HPV DNA testing, which alone is inadequate for predicting which infections may lead to cancer. These findings highlight the importance of refining diagnostic tools to effectively triage women at risk and minimize unnecessary medical procedures (31–34).

In this study, we assessed the methylation status of the HPV16 L1 gene using quantitative pyrosequencing on exfoliated cells gathered from Pap tests. This technique is crucial for identifying individuals at increased risk of rapid cancer progression. The DNA from exfoliated cells offers a practical alternative to DNA from formalin-fixed paraffin-embedded sections, which, although providing accurate methylation levels, are not suitable for mass screening. Studies have confirmed that methylation profiling can effectively discriminate between normal or transient HPV infections and more severe conditions, such as CINs 2 and 3 and cervical cancer (32, 33). It has been found that methylation levels in host genes are generally lower than those in the HPV16 L1 gene, suggesting a specific pattern that could be critical for enhancing screening and early detection methods.

In the examination of methylation levels of the HPV16 L1 gene in cervical cancer cell lines such as CaSki and SiHa, studies have confirmed high methylation regardless of the copy number of integrated HPV16. This aligns with previous research. This methylation, typically observed in the L1 gene of HPV16, is considered to result from the cellular transformation processes that occur once HPV is integrated into the host genome (35, 36). This ongoing methylation of HPV16’s late genes is crucial as it progresses during carcinogenesis, indicating a potential role for these methylation markers in both the diagnostics and prognostics of cervical cancer. The present study demonstrates high methylation levels in the HPV16 5’L1 gene, particularly at CpG sites 5927 and 5963, whereas very low methylation levels were observed in the 3’L1 gene. These specific hypermethylated CpG sites have been previously reported to differentiate between normal and cervical neoplasia, consistent with our findings. Other CpG sites, such as 6365 (6367 in this study), have been identified as strong predictors for CIN2 progression, though they were not analysed in the present study. According to the results presented in this study, methylation levels at position 6367 are high in cervical cancer. Methylation levels at position 6367 were significantly different between cervical cancer and inflamed cells. In the ROC curve analysis, the 6367 locus showed high diagnostic accuracy and was able to better distinguish high-grade cervical lesions from milder conditions. In summary, the gene locus at position 6367 has important features in the methylation pattern of cervical cancer, and high accuracy shown in diagnosis make it a potential biomarker for early diagnosis of cervical cancer. Hypermethylation of the HPV16 L1 gene has also been associated with HPV-related diseases, including vulvar intraepithelial neoplasia, oral carcinomas and penile carcinoma.

The association between the hypermethylation of the HPV16 L1 gene and the integration status of HPV16 into host chromosomes is supported by several studies (35, 37, 38). This hypermethylation is correlated with the overexpression of the E6 and E7 oncoproteins. Specifically, the overexpression of E7 is known to activate DNA methyltransferase 1, leading to the further methylation of HPV16 late genes. This relationship has been observed not only in HPV16 but also in other high-risk HPV types, including HPVs 18, 31 and 33, all of which show significant hypermethylation in their L1 and L2 regions associated with CIN3 conditions. Moreover, comprehensive methylation assessments have proven more effective than traditional HPV16 and HPV18 genotyping in identifying advanced cervical lesions, reinforcing the diagnostic and prognostic value of these methylation markers (27).

The methylation of the long control region (LCR) in the HPV16 genome has been explored as a potential biomarker for cervical cancer, although studies have shown varied results. Some research indicates that the LCR region can be hypomethylated or unmethylated in cases of cervical cancer, suggesting a lack of consistent methylation patterns (39). Conversely, other studies report moderate to high methylation levels in this region within cervical cancer samples (40). These discrepancies may reflect adaptive changes in the viral genome under selective pressure, influencing viral replication and integration and subsequently affecting the epigenetic regulation of promoter activity (41). In this study, we observed high methylation levels in the HPV16 5’L1 region in carcinoma samples. The ROC curve analysis demonstrated high sensitivity and specificity when 20%–30% methylation was used as the cutoff point at CpG site 5927 and 15%–25% as the cutoff point at CpG site 5963. These results suggest that L1 gene methylation percentages could serve as effective biomarkers for predicting the risk of rapid progression to cervical cancer during HPV-induced transformation. Combining CpG sites 5927 and 5963 may offer the best predictive accuracy, as this combination increases specificity. Our findings indicate that methylation levels at specific CpG sites within the HPV16 L1 gene can serve as reliable biomarkers for the early detection and prognosis of cervical cancer. High methylation levels, particularly at CpG sites 5927 and 5963, are strongly associated with advanced cervical lesions. These sites hold promise for clinical screening and diagnostic protocols, potentially reducing the need for unnecessary colposcopies and allowing for more targeted follow-up.

Future studies should focus on validating these findings in larger, more diverse cohorts to establish the robustness and generalizability of these biomarkers. Additionally, exploring the combination of HPV16 L1 gene methylation with other genetic and epigenetic markers could enhance the predictive power of these assays. Ultimately, integrating these biomarkers into existing cervical cancer screening programs could improve early detection and patient outcomes, particularly in high-risk populations such as the Uygur and Han women studied herein.

In this study, DNA methylation-based methods showed high specificity and sensitivity in detecting cervical cancer and precancerous lesions. Techniques for DNA methylation can complement HPV16 and HPV18 genotyping and have improved performance compared with widely used cytological tests. Methylation testing, as a screening tool, can be used as a candidate diversion method in areas lacking trained cytology professionals to provide feasible follow-up solutions for areas where colposcopy is not readily available. However, some limitations remain in this study. First, the limited nature of the study’s sample size may affect the generalizability of its results. We will subsequently collect more clinical samples to confirm the validity of the conclusions. In addition, the study focused on specific populations or regions, further limiting the broad applicability of its results. Finally, HPV16 methylation status is closely associated with cervical lesion progression, and its molecular mechanism requires further investigation.

## 5 Conclusion

In conclusion, this study supports the potential of HPV16 L1 gene methylation as a biomarker for cervical cancer progression. The specific hypermethylation of CpG sites 5927 and 5963 provides a strong basis for further research and clinical application. The CpG site 6367 has important features in the methylation pattern of cervical cancer, and high accuracy shown in diagnosis make it a potential biomarker for early diagnosis of cervical cancer. These findings contribute to the growing body of evidence on the role of epigenetic modifications in HPV-related carcinogenesis and highlight the importance of developing precise and effective screening tools to combat cervical cancer.

## Data Availability

The original contributions presented in the study are included in the article/supplementary material. Further inquiries can be directed to the corresponding author.
